# The Medical Practice Law under Fire

**Published:** 1903-03

**Authors:** 


### TEXAS MEDICAL JOURNAL.

AUSTIN, TEXAS.

A MONTHLY JOURNAL OF MEDICINE AND SURGERY.

EDITED AND PUBLISHED BY

F. E. DANIEL, M. D.

ASSOCIATE EDITORS:

WITTEN BOOTH RUSS, M. D., San Antonio, Texas.

P. M. PAYNE, M. D., Brownwood, Texas.

Published Monthly at Austin, Texas. Subscription price $1.00 a year in advance.

Eastern Representative: John Guy Monihan, St. Paul Building, 220 Broadway, New York City.

Official organ of the the West Texas Medical Association, the Houston District Medical Association, the Austin District Medical Society, the Brazos Valley Medical Association, the Galveston County Medical Society, and several others.

### THE MEDICAL PRACTICE LAW UNDER FIRE.

**"The Drugless Doctors."**

Senate bill No. 175 was introduced into the Senate February 19th, and was reported upon favorably by the Committee on Public Health. It came up for consideration February 25th, and there was a two days furious fight upon it. It is still pending at this writing, March 2nd, and is to be taken up again tomorrow. It is called the "Doctors' Bill." It seeks to remedy certain defects in the law, chief of which is that senseless, unjust and unconstitutional clause that exempts from its provisions all "those who do not give or prescribe drugs or medicines." The fight is on the proposition to strike out that clause. An amendment to the bill offered by Hanger of Fort Worth and McKamy of Dallas, who are championing and defending the quacks, reads: "Provided, the provisions of this act shall not apply to those persons who do not give or prescribe drugs or medicines." It will be seen that this is the exact wording of the law as it now stands on the statute books, passed at last session and made effective July 9, 1901.

If this amendment carries, the law will remain unchanged except in a few minor and unimportant details. In the cause of legitimate medicine; in the interest of the public safety; because of right and justice, this odious amendment should be and must be, and I firmly believe will be, defeated. The Constitution of Texas says: "The State shall have power to regulate the practice of medicine, but no preference shall be shown any school of medicine." (There are no "schools of medicine, they are only sects and pathies—abominations and absurdities. Still, the State recognizes them as "schools.") Are these persons not practicing medicine within the meaning of the law? The supreme courts of many States have decided that they *are*. Is not a "preference" being shown these drugless fellows by the State in exempting them from the requirements to which all other practitioners are compelled to conform? It is, therefore, unconstitutional as well as unjust, unsafe and absurd.

Just before the discussion opened a copy of the following letter was put into the hands of every Senator, and all the audience. The audience was largely made up of "Christian science" fanatics, male and female; homeopaths, osteopaths, magnetic healers (whatever that may be), the healer with an Indian assistant who has an X ray eye and can see your insides and diagnose disease,—vitopaths, physio-medico-paths, representatives of the regular medical profession and others:

THE PUBLIC HEALTH AND SAFETY.

THE "DRUGLESS DOCTORS."

*To the Senators:*

In Monday's *Statesman,* under the head of "Legislative Gossip: Drugless Doctors May be Debarred," occurs the following statement, which is untrue and misleading. The reporter says: "The real purpose of the bill is to prohibit drugless doctors from practicing their profession in Texas." I deny it. The real purpose of the bill is to compel them to comply with the law and procure a license from the State, in the same way that all other practitioners are required to do. The young men educated in the Medical Department of our own University and given the medical degree are still required to stand examinations before our State Board of Examiners, and so are all other graduates in medicine. It is a discrimination against graduates of medicine, and especially our young men, that a crank, an irregular, a graduate of nowhere and in nothing, should be exempt from examination and permitted ignorantly to exercise a privilege so fraught with danger to the public health and safety. It is a stultification that the State requires its educated and trained men to stand examination and thus procure a license, and permits the ignorant and uneducated to practice without either.

Protection of the public health is the State's highest duty. Upon the health and strength of the people depend the prosperity, the integrity, the perpetuity of society, the State and the nation. It must be safeguarded from all sources of danger, not only diseases, but accidents and injuries. The causes of disease can be largely removed by sanitary science; restrictions must be put upon all pursuits and occupations that are dangerous to the health or life of the citizen. The practice of medicine is a dangerous pursuit at best, but when the privilege of practicing medicine is entrusted to an ignorant or unscrupulous person, that person is licensed *to deal with life and death.* Not only is the health of his "victims" in his hands, but the life. The ignorant "doctor" is a menace—a perpetual danger—that should be guarded against. That a man has the right to select his own doctor may be true, but the State should see that *there are only qualified doctors so select from;* the public do not know the difference between a quack and a qualified physician. A man could as truthfully say that he has a right to travel on a passenger train in charge of a blind engineer. The State should safeguard this dangerous privilege. No man should be permitted by the State to deal with the health and life of its citizens who can not show to its legal authorities that he has received the necessary schooling, training and instruction to qualify him to intelligently exercise the privilege. Examination on the several branches of medicine should be the only test. The practice of medicine does not alone consist of giving drugs, as the amenders of the act at last session seemed to believe. The practice of medicine is the treating of disease or injury by any means or methods, material or immaterial. The supreme courts both of Illinois and Alabama have recently ruled that an "osteopath" (whatever that may be) is "practicing medicine" within the meaning of the statutes, and must come under the laws regulating the practice. A "Christian scientist" (whatever that may be) is "practicing medicine." Were these people educated in anatomy, physiology, therapeutics, chemistry, hygiene, etc., and could show to the State's satisfaction that they *are,* no one would care what "methods" they followed in practice, whether they gave drugs or not. But their sins are sins of omission as well as of commission. To illustrate both: A citizen is deluded by the belief that "Christian science" can cure diphtheria, for instance, by prayer. Such person allows one of those fanatics to pray over his child until it dies. That child should have the benefit of skilled medical treatment, and it is permitted to die for the lack of it. Nothing is better demonstrated than that by timely and proper treatment the mortality of that once dread scourge has been reduced from about 54 per cent. to about 4 or 4½. In St. Louis not long ago this very thing happened in the family of a rich man. He was indicted for contributory negligence and was made accessory to the death of his child and fined heavily. A man may claim the right to commit suicide by this or any other method, but he should not be permitted to kill his child. Take a case of sin of commission: Near Austin an irregular practitioner was called to see a child with a fracture of the bone extending into the shoulder joint. *He pulled on it* at intervals for several days, saying the shoulder was out of joint. The child, in a horrible condition, was brought to a physician in Austin and its life saved. Does any rational man contend that such persons as these should be entrusted by the State with so dangerous a privilege and exempted from the operation of the law? It is madness. No such person should be licensed (or permitted, without license) to tamper with the health and life of the citizen. The privilege of practicing medicine, I repeat, should be granted only to those who can show to the State that they are fitted by such study, experience and instruction to exercise it. Otherwise, the State is virtually permitting the ignorant in many cases to TAKE LIFE.

Very respectfully,

F. E. Daniel, M. D.,

Editor Texas Medical Journal.

Hot Shot.—Senators Davidson of DeWitt, Hicks of Bexar, Savage of Montague, Douglas (Dr.) of Hill, Perkins of Cherokee and Cain of Raines shelled the woods and created consternation in the camp of the drugless ones and their red-headed sympathizers who were present and prayerfully and tearfully beseeching certain Senators to remember that the Christian science disciples at least, were healing the sick by divine assistance and inspiration (taking pay for it all samee like a doctor). One Senator who was wavering and inclined to vote for the amendment, after having been cajoled and talked to by a sister, said that one more experience of the kind would make him vote for a still stronger prohibition—and suggested an asylum or jail. Hicks, Savage, Davidson, Perkins, Douglas and Cain certainly did show up the Christian science craze in a most ridiculous light, and made the strong points that the exemption of the drugless ones was unconstitutional, unjust, dangerous to the public health and absurd on the face of it. I want every reader of this to remember all his life the splendid battle waged by these enlightened and earnest gentlemen in the cause of legitimate medicine and the public safety. All honor to them! And I want, also, that no physician worthy of the name shall ever forget that Senators Hanger, McKamy, Grinnan, Hale and Henderson defended the drugless ones in the face of the facts made evident, that they were allowed an exemption from the requirement that the State puts upon the educated physician—(examination and license to practice)—in clear violation of the Constitution. The debate was "better than a circus." The last mentioned gentlemen made eloquent speeches, worthy of a better cause. I hope they will yet have their eyes opened to the true state of the case, and that they will vote to kill the amendment that seeks to perpetuate this exemption of all the pathics and fanatics—thus licensing them to kill with impunity. The fight will be resumed by the time this goes to press, and doubtless a vote will not be reached before our forms are closed.

"A Nigger in the Wood Pile."—It is just *too* funny. The Senate Committee on Public Health met to consider the State Medical Association Committee's bill—the "Public Health Bill," and invited Drs. Daniel and Tabor, of the committee, to address them, which they did, and the bill was reported favorably. There was present one "Doctor" Davis, of Austin, by whose invitation no one knows, if any. After Drs. Daniel and Tabor had addressed the committee, without invitation this party got up and said: "Gentlemen, I represent the drugless side of the medical profession." Dr. Daniel asked what that was. He said: "Christian science, osteopathy, magnetic healers, and all who do not give drugs." Dr. Daniel asked: "Which are you?" "A magnetic healer," he said; and continued: "Gentlemen, there's a nigger in the wood pile, and I want to show him to you. Dr. Daniel has been talking very learnedly about bacilli and germs causing consumption; I want to tell you that there are no such things, and all diseases are caused by heat or cold." Dr. Daniel asked: "Which causes consumption?" "Cold," he said. Dr. Daniel begged to interrupt the "doctor," and said, addressing the committee: "Gentlemen, I should introduce this person to you, as none of you seem to know him. This is Dr. Davis, the magnetic healer of Austin. He has a partner, a negro, or a Mexican, or an Indian (which is he, doctor?) who has an X ray eye, and can see through you gentlemen, or anybody else, and tell you what's the matter with you. He diagnoses the doctor's cases, and can see the "cold" which the doctor says causes consumption. Is that not so, doctor?" "Yes," he said.

The doctor was then asked to show up the nigger, and pulling out an old copy of the *Statesman,* read extracts from Dr. Daniel's Dallas address on "The Paramount Duty of Texas to the People." He said: "Gentlemen, you will see that he says *we* are all *nuisances.*" "Well, what of it?" asked a Senator, "where's the 'nigger in the wood pile?'" "Why, don't you see him?" said the excited magnetic healer,—"*here* he is, in this *bill,* (reads): 'The State Health Officer shall have authority to abate *nuisances.*' He wants to abate *US,* Sirs!"

And that fellow is permitted to practice medicine without license, let or hindrance. What a commentary on the intelligence of the legislators who exempted the drugless ones!

---

THE DAY BEFORE DR. JOHNSON'S BILL (see "News and Miscellany") came up in the House, the following editorial appeared in the *Austin Daily Statesman* (February 18th). Some of the "Red Back's" readers may recognize the tone of voice:

RAILWAY CAR SANITATION.

A bill has been introduced in the Legislature authorizing the State Health Officer to require railroad companies to disinfect their sleeping cars and day coaches. The bill was drafted by Dr. Johnson, of Lee county. It is learned that it has been reported favorably by both the Senate and House committee with the recommendation that it do pass.

This is a very important measure, and should it become a law it will do a great deal toward protecting the public from a scarcely suspected danger, but one compared to which yellow fever, smallpox and cholera are insignificant. The *Statesman* asserts upon reliable authority that the deaths from consumption alone—a disease easily preventable—are, as compared to yellow fever, as 150 to 1. That is, while one hundred thousand persons have died of yellow fever in the United States within the last one hundred years, one hundred and fifty thousand persons die of consumption in the United States every year. And yet, in Texas, all the health machinery of the State is directed solely to keeping out yellow fever, smallpox and bubonic plague, and all of the money appropriated for sanitary purposes is expended for quarantine and inspection service.

Consumption is not contagious, but is rapidly and fatally communicable from the coughing invalid to well persons. The poison is the tubercle bacillus, a microscopic organism, which is coughed up and spit out. When it falls on the floor, furniture or furnishings of a room or car, it dries and is mixed with the dust, which is inhaled by well persons.

The poisonous bacilli rapidly prolifuate in the lung and a swarming colony of these micro-organisms soon appears and the deposit of tubercle in the lung is the result.

The micro-organism is very tenacious of life, and can only be destroyed by fire or by certain strong chemicals, such as bichloride of mercury and carbolic acid, which should be put in all the spittoons; and when lodged in curtains, blankets, carpets, upholstered furniture, etc., where it can not be treated by these chemicals, it can only be reached and killed by the well known germicidal gases, formaldehyde, sulphur dioxide, etc.

Exposed to the direct rays of the sun every day, the germ has been found alive at the end of three years in a room which had formerly been occupied by a consumptive patient. It will thus be seen how absolutely necessary it is for the protection of the traveling public that railway cars and hotels and boarding houses should be subjected to the only known means of preventing consumption—disinfection; and the matter should be under the direction of the public health department, just as in the case with infected vessels from foreign countries.

The State Health Officer, under existing laws, causes the inspection of every vessel entering any Texas port, and if one be found to be infected with any disease dangerous to the public health, or even suspected of being infected, he puts it in quarantine and subjects it to a sanitary policing, including fumigation, all of which is done at the cost of the vessel, the fees being fixed by law.

There can be no good reason why the State should not require the same supervision of all railroad trains entering the State, but many reasons why it should; and, indeed, under existing laws, the State Health Officer can stop any train at the State borders, and if infected or suspected of being infected, he can compel disinfection, or refuse admission of train passengers or crew into the State. Why, then, should he not enforce the same precautions within the State?

The general public are as innocent of knowledge of the dangers of infected sleeping cars and hotel rooms as are children. It is almost criminal to permit them to be thus ignorantly exposed to contagion. It is well known that the negro porters of sleeping cars often acquire the disease from the dust of the cars, and the mortality among that class is very heavy.

This legislation is by no means aimed at the railroads, but is really in their interest; but primarily it is in the interest of the public health, and it is the State's first and highest duty to safeguard that.

One of the big railroad companies recently had to pay—upon final decision of the Supreme Court of Texas—the sum of $15,000 damages to the family of a passenger who contracted smallpox in one of their cars.

Fumigation and disinfection, while protecting the public from infection with consumption, smallpox, scarlet fever, diphtheria, etc., will protect the railroad company from damage suits. Should suit for damages be brought, the fact that the cars had been disinfected exactly as directed by the State health authorities, would insure defeat of the suit. And when one railroad begins to sterilize its cars and advertises the fact, other companies will have to do the same, whether made to do it by law or not, or lose the passenger traffic. The *Statesman* can not see how any one, especially the railroads, can reasonably object to this very necessary measure of protection both of the public health and of their own interests. It is said that as compared with the disinfection of a ship the cost of car fumigation and policing will be utterly insignificant. The bill should become a law, and the *Statesman* sincerely hopes that it will.

---

A VOTE on what I call the anti-quack amendment to the medical practice act (Senate bill No. 175), having been staved off until the absentees can be rounded up—the friends of legitimate medicine and the public safety sent a copy of the following letter to every regular physician in Texas meantime. Today is Texas Day, and a legal holiday (March 2nd), and there's "nothing doing." The bill will be taken up tomorrow, and there will be firing all along the line. We will win out in the Senate I'm sure, on a showdown; and then it will all have to be fought over in the House. Friends of the cause of right (that all who practice medicine should comply with the law, see "News and Miscellany" elsewhere) are expected and urged to fire in telegrams and letters to their representatives right away. One fellow in the Senate who is championing the quacks, got seventeen telegrams in about seventeen minutes. He looked mighty tired:

AUSTIN, TEXAS, February 28, 1903.

*Dear Doctor:*

A bill has been introduced in the Senate by Davidson of DeWitt and Hicks of Bexar, known as Senate bill No. 175, which embodies some necessary amendments recommended by the Boards of Medical Examiners to perfect the new medical law passed two years ago by the Legislature. There is no opposition to any of these amendments, except the one which requires those parties "who do not use drugs or medicines" to undergo an examination testing their qualifications, the same as the members of the regular, homeopathic and eclectic schools. Or, in other words, requiring them to submit to tests the same as are required of all young men of this State who undertake to be competent, qualified physicians. This bill came up in the Senate as a special order on Wednesday, February 25th. An amendment was introduced by Senators McKamy and Hanger, which is now pending, and if adopted would leave the bill as it now stands.

By means of active work the friends of medical legislation have been able to keep the Senate from a vote on the question. The Senate has adjourned until 10 o'clock on Tuesday, March 3rd, then this bill with amendment is made a special order, and must come to a test vote. While we feel reasonably certain of success, still we want a few more Senators on our side of the question, in order to be absolutely certain. The Senators, so far as we are able to ascertain, who oppose the bill are: J. T. Beaty, J. J. Faulk, Arch Grinnan, J. M. Hale, W. A. Hanger, J. L. Harbison, A. J. Harper, Travis Henderson, J. W. Hill, A. G. Lipscomb, R. W. Martin, W. C. McKamy, Seth P. Mills, A. W. Morris and James Patteson. If there is one or more of this list whom you might influence, either by personal interview, telephone or telegraph message, or through political or personal friends, urging them to vote down the McKamy and Hanger amendment, and support the bill introduced by Davidson and Hicks, we earnestly urge you to do so. Do not lay this letter aside, but devote every spare moment of your time in assisting us to rid the State of this class of incompetents. If we succeed in our efforts next Tuesday in the Senate, the whole matter will be referred at once to the House of Representatives, and it is equally important that you begin to influence the members of the House in every possible way to give their earnest, uncompromising support to this measure, without any amendments or substitutes. Many of us are now devoting much of our time in looking after the interests of this measure, and we feel sure you will contribute your part towards the work. Trusting your Senator and Representatives will vote right, we remain,

Yours fraternally,

M. M. Smith,
Secretary of Board of Medical Examiners.

F. E. Daniel,
Chairman of Committee on Public Health, State Medical Association.

Bacon Saunders,
Ex-President Texas State Medical Association.

A. Garwood,
Ex-President Western Texas Medical Association.

T. J. Bennett,
Ex-President Austin District Medical Society.

And Others.

